# Green Synthesis of Silver Nanoparticles by *Cytobacillus firmus* Isolated from the Stem Bark of *Terminalia arjuna* and Their Antimicrobial Activity

**DOI:** 10.3390/biom11020259

**Published:** 2021-02-10

**Authors:** Sujesh Sudarsan, Madan Kumar Shankar, Anil Kumar Belagal Motatis, Sushmitha Shankar, Darshan Krishnappa, Chakrabhavi Dhananjaya Mohan, Kanchugarakoppal S. Rangappa, Vijai Kumar Gupta, Chandra Nayaka Siddaiah

**Affiliations:** 1 Department of Studies in Biotechnology, University of Mysore, Manasagangotri, Mysore 570006, India; sujeshmayyil325@gmail.com (S.S.); anilkumarbm0908@gmail.com (A.K.B.M.); sushmithagangatkar@gmail.com (S.S.); devdarshan549@gmail.com (D.K.); 2 Institute of Excellence, VijnanaBhavan, University of Mysore, Manasagangotri, Mysore 570006, India; madan.mx@gmail.com; 3 Department of Studies in Molecular Biology, University of Mysore, Manasagangotri, Mysore 570006, India; cd.mohan@yahoo.com; 4 Department of Studies in Chemistry, University of Mysore, Manasagangotri, Mysore 570006, India; rangappaks@gmail.com; 5 Center for Safe and Improved Food, Scotland’s Rural College (SRUC), Kings Buildings, West Mains Road, Edinburgh EH9 3JG, UK; 6 Biorefining and Advanced Materials Research Center, Scotland’s Rural College (SRUC), Kings Buildings, West Mains Road, Edinburgh EH9 3JG, UK

**Keywords:** *Terminalia arjuna*, endophytes, *Cytobacillus firmus*, silver nanoparticles, antimicrobial activity

## Abstract

This work reports an eco-friendly synthesis of silver nanoparticles (AgNPs) using endophytic bacteria, *Cytobacillus firmus* isolated from the stem bark of *Terminalia arjuna*. The synthesis of AgNPs was confirmed by visual observation as a change in color of the bacterial solution impregnated with silver. Further, the morphology of the AgNPs, average size, and presence of elemental silver were characterized by UV–Visible spectroscopy, scanning electron microscopy, and dynamic light scattering spectroscopy. The roles of endophytic secondary metabolites in the metal reduction, stabilization, and capping of silver nanoparticles were studied by qualitative FTIR spectral peaks. The antimicrobial ability of AgNPs was evaluated against Gram-positive (*Staphylococcus aureus)* and Gram-negative (*Escherichia coli)* bacteria and pearl millet blast disease-causing fungi (*Magnoporthe grisea*). The biosynthesized AgNPs showed good antibacterial and antifungal activities. AgNPs effectively inhibited the bacterial growth in a dose-dependent manner and presented as good antifungal agents towards the growth of *Magnoporthe grisea.*

## 1. Introduction

Plants are home to a large number of symbiotic and non-symbiotic microorganisms, and these microbes are known to exhibit noteworthy roles in crop development, protection, and growth [1]. The relationship between plants and microbes is imperative for their existence in a stressed environment [2]. The organisms that reside in the tissues of a plant are known as endophytes [3,4]. A large number of attempts have been made in the previous decade to explore the biological applications of endophytic organisms, and these microbes have been widely utilized for the synthesis of nanoparticles. Nanoparticles are chemical entities with a size below 100 nm with various biological applications, and they can be prepared using physical, chemical, and biological methods [5]. Physical and chemical methods comprise synthesis by laser ablation, evaporation-condensation, chemical reduction, microwave, sol-gel process, vapor deposition, and laser pyrolysis [5,6,7]. Although physical and chemical methods have gained significant popularity over biological methods, their biomedical applications are greatly limited due to the use of toxic and hazardous chemicals. Biological methods utilize bacteria, plant extracts, yeasts, and fungi which are known to captivate and gather metals that can be utilized as reducing agents and regulate the nanostructure topography of the metal ions [8]. Among biological agents, microbe-mediated synthesis of nanoparticles remains the mainstay. Microorganisms have been known to be vital nano-factories that hold enormous potential for the synthesis of nanoparticles due to their catalytic efficiency, eco-friendliness, cost-effectiveness, and lesser toxicity. Microorganisms have various cellular reductases, which are known to reduce metal salts to metal nanoparticles with a narrow size distribution, and therefore, less polydispersity. Microbial enzymes and secondary metabolites released by microorganisms play a strategic role in the transformation processes of metal ions into their corresponding nanoparticles. It has been observed that, when the microbes are exposed to unfavorable conditions (such as exposure to a metal ion solution), they release enzymes and metabolites that possess reducing potential, by which the metal ions could be converted to metal nanoparticles [9,10]. The potential of endophytic organisms to convert the metal salts to their corresponding nanoparticles has been studied extensively. The culture supernatant, bacterial biomass, and bacteria-derived components can be used for the synthesis of nanoparticles. Endophytic fungi are known to be ideal for the synthesis of nanoparticles, as they can easily develop large biomasses within a short period. The endophytic fungi can endure agitation, low pressure, and the conditions of a bioreactor. Endophytic micro-organisms generate biologically important substances having applications in agriculture, modern medicine, and other pharmacological areas [11]. Previously, endophytic bacteria including *Bacillus cereus*, *Bacillus persicus,* and *Bacillus licheniforms* have been used for the production of nanoparticles [12,13].

Silver is a potent antimicrobial agent with a wide number of in vitro and in vivo benefits, especially for humans [14]. Topical dressing with silver is commonly used in the treatment of open wounds and chronic ulcers [15]. Silver nanoparticles (AgNPs) have been known to possess prospective benefits in many areas, such as antibacterial, drug delivery, biological sensors, filters, textiles, etc. [16,17]. Various parameters such as temperature, pH, method of synthesis, incubation time, and type of biological substances impact the shape, size, and activity of the AgNPs synthesized [18]. We have used extracellular contents of *Cytobacillus firmus* for the synthesis of AgNPs. This method has several advantages over the intracellular counterpart, as it is devoid of sonication, repeated centrifugations, and washing procedures [9].

In the present study, we have isolated the endophytic bacteria from *Terminalia arjuna* and identified them as *Cytobacillus firmus* using 16S rRNA and used *Cytobacillus firmus* for the synthesis of AgNPs. The new AgNPs showed good antimicrobial activity against reference bacterial (*Staphylococcus aureus* and *Escherichia coli*) and fungal (*Magnoporthe grisea*) strains.

## 2. Materials and Methods

### 2.1. Collection of the Bark Sample of Terminalia arjuna

The bark sample was collected by skinning the bark of *Terminalia arjuna* from the Chandravana, a medicinal plant garden, Mysore, Karnataka, India. The sample was cut into small pieces (15–20 cm in length × 3–5 cm in width × 1–2 cm in height) for further study in the laboratory.

### 2.2. Isolation and Identification of Endophytic Bacteria

The bark sample was surface sterilized by dipping it in 96% alcohol and 1% sodium hypochlorite solution for one minute and five minutes respectively. It was again re-immersed in 70% alcohol and passed through the flame followed by cleaning with sterile distilled water (4 times). Bark samples were shade dried, and 90 g of bark sample was crushed using the mixer, and 10 mL of 0.85% sterile NaCl solution was added. The crushed sample was used as the source of endophytic bacteria. Isolation of endophytic bacteria was performed by a serial dilution (10^−2^ to 10^−4^); 100 µL of bacterial suspension from 10^−3^ and 10^−4^ were added onto Petri plates containing nutrient broth and spread and incubated the plates at 37 °C until the growth of the endophytic bacteria colony was visible. Pure culture of individual colonies was isolated from the obtained bacterial population, and morphological identification of bacteria was done by gram staining method.

### 2.3. Molecular Identification of Endophytic Bacteria

The endophytic bacteria were identified by amplification and sequencing of the 16S rRNA region. The extraction of genomic DNA was performed by the CTAB method. The amplification of the 16S rRNA region of endophytic bacteria was performed with two universal primers 27F (5′-AGA GTT TGA TCC TGG CTC AG3′) and 1492R (5′-CGG TTA CCT TGT TAC GAC TT-3′) in a conventional PCR (Veriti 96-well thermal cycler, Applied Biosystems). The conditions for amplified gene fragment were initial-denaturation of the target DNA at 94 °C for 5 min followed by final denaturation at 94 °C for one min, primer annealing at 55 °C for one min, initial extension at 70 °C for 2 min, and a final extension at 72 °C for 10 min. The PCR was performed with a total reaction volume of 25 µL containing 196.2 ng of DNA template, 1 µL each of both 10 µM forward (27F) and reverse primer (1492R), 10.5 µL of nuclease-free water, and 12.5 µL of PCR master mix [19]. PCR was set to 30 cycles. PCR product was visualized by gel electrophoresis using 1.2% agarose gel stained with ethidium bromide. The size of the PCR product was distinguished with the help of a 100 bp to 10 Kb ladder (Genei lot number: MBD292098) by applying an electric voltage of 80 V for 90 min. The result of electrophoresis was observed by a UV Light Gel Documentation System (BIORAD Gel doc XR + system, (BIO-RAD, Berkeley, CA, USA). Sequencing was performed by Barcode Biosciences, Bangalore using Sanger sequencing big dye terminator v3.1 cycle sequencing kit (Thermofischer scientific, Waltham, MA, USA). For blast searches and species identification, the resources of the National Center for Biotechnology Information (NCBI, https://www.ncbi.nlm.nih.gov/) (accessed date: 18 January 2021) was used.

### 2.4. Screening of Endophytic Bacteria for the Synthesis of Silver Nanoparticles

Endophytic bacteria were cultured in nutrient agar media incorporated with silver nitrate (1 to 5 mM) and incubated at 37 °C until visible growth was observed. After understanding that the bacteria can grow up to the concentration of 5 mM of silver nitrate, the bacteria were used for the synthesis of AgNPs. The bacteria were inoculated into 100 mL of nutrient broth and incubated in a rotary shaker incubator at 120 rpm at 37 °C for 24 h. 10 mL of silver nitrate having concentrations of 1 to 5 mM was added into 90 mL of bacterial culture in five different conical flasks and incubated until the color change from white to yellowish-brown. Samples were drawn periodically and monitored using UV–Visible spectrophotometry at 200–800 nm to confirm the synthesis of nanoparticles.

### 2.5. Physical Characterization

#### 2.5.1. UV–Visible Spectroscopy

The absorbance spectrum of the colloidal sample was analyzed in the range of 200–800 nm, using a UV–Visible spectrometer Shimadzu-UV 1800 (Shimadzu, Kyoto, Japan) with nutrient broth as a reference. Further FTIR analysis was carried out to differentiate the biomolecules in the endophytic bacteria which were accountable for the reduction of silver and the stabilization of nanoparticles.

#### 2.5.2. FTIR Spectroscopy

FTIR analysis was performed by using a PerkinElmer Fourier-transform infrared spectrometer (Spectrum two, Perkin Elmer, Waltham, MA, USA) with a resolution of 4 cm^−1^ in the range of 400–4000 cm^−1^. A lyophilized semisolid AgNPs sample was used for the spectroscopic analysis.

#### 2.5.3. Fluorescent Microscopy

Fluorescent microscopy was done by taking 1 mL of the bacterial suspension (10^7^to 10^8^ CFU per mL) and AgNPs (2 times of Minimum inhibitory concentration) and mixing them in a centrifuge tube followed by incubation at 30 °C for 12 h with gentle mixing. After harvesting through centrifugation at 3000 rpm for 10 min at 4 °C, the bacteria were re-suspended in 500 µL of chilled 1× PBS and the cells were stained with 1µL of acridine orange (AO, excitation/emission at 535 nm/617 nm; Sigma-Aldrich) for 15 min and counterstained with 1 µL of ethidium bromide (EB, excitation/emission at 358 nm/461 nm; Sigma-Aldrich) for 5 min in the dark. The cells were centrifuged again at 3000 rpm for 10 min at 4 °C followed by washing with 500 µL of 1× PBS. The cells were then uniformly spread onto a glass slide cleaned with ethanol and viewed under Carl Zeiss Axio vision 3.0 fluorescent microscopes (Carl Zeiss, Jena, Germany).

#### 2.5.4. Scanning Electron Microscope (SEM)

A lyophilized sample of AgNPs was subjected to Zeiss EVO-18 scanning electron microscope (Carl Zeiss, Jena, Germany) at 20 kV to study the morphological features of AgNPs at magnifications 6000×, 8000×, and 10,000×.

#### 2.5.5. Dynamic Light Scattering (DLS)

The size distribution and exact sizes of nanoparticles in the colloids were measured using a Microtrac wave-particle size analyzer (Microtrac, Montgomeryville, PA, USA). The measurement gave the average hydrodynamic diameter of the nanoparticles and the zeta potential analysis (polarity, viscosity, conductivity, charge, pH, etc.). All measurements were performed in triplicate with a temperature equilibration time of 1 min at 25 °C. The data processing mode was established to high multi-modal resolution. The semisolid suspension of the synthesized AgNPs was diluted 4-fold and the diluted sample was allowed to filter through a 0.22-μm syringe driven filter and given elucidation using DLS equipment (Microtrac Nano trac flex, Microtrac, Montgomeryville, PA, USA).

#### 2.5.6. XRD Analysis

The synthesized AgNPs were centrifuged at 10,000 rpm for 15 min and the pellets were dispersed in sterile double distilled and centrifuged at 10,000 rpm for 10 min. The purified pellets were dried at 50 °C in an oven and analyzed by an X-ray Diffraction Unit (XRD) (Proto AXRD Benchtop, Los Angeles, CA, USA). The X-ray diffraction (XRD) measurement of AgNPS synthesized by endophytic bacteria was carried out using a Cu-K*α* radiation source in a scattering range of (2θ) 5–70 on the instrument operating at a voltage of 30 kV and a current of 20 mA. The presence, crystalline nature, phase variety, and grain size of synthesized AgNPs were determined by X-ray diffraction spectroscopy. The particle size of the prepared samples was determined by using Scherer’s equation as follows:

$$D=\frac{K\lambda }{\beta .\;cos\theta }$$

where *D* is average crystallite size and *β* is line broadening in radians (full width at half maximum of the peak in radians). *K* is the wavelength of X-ray (*λ* = 0.154 nm) and *θ* is Bragg’s angle. *K* is constant (geometric factor = 0.94).

### 2.6. Biological Activity of Silver Nanoparticles

#### 2.6.1. Antibacterial Assay

The antibacterial activity of AgNPs against *Escherichia coli and Staphylococcus aureus* was evaluated using the Kirby Bauer antibiotic disc diffusion assay, the broth micro-dilution method, and minimum bactericidal concentration (MBC). The nutrient medium was used to subculture bacteria and was incubated at 37 °C for 24 h. Overnight fresh cultures were collected and spread on nutrient agar plates to grow bacteria. Sterile discs (5 mm) were saturated with double distilled water and streptomycin as negative and positive controls, respectively. Different concentrations of AgNPs (5, 10, and 20 µg/µL) were added onto the discs, and the plates were incubated at 37 °C for 24 h. Antibacterial activity was measured based on the zone of inhibition around the disc impregnated with double distilled water, streptomycin, or AgNPs. The minimum inhibitory concentration (MIC) and minimum bactericidal concentration (MBC) of green synthesized AgNPs were performed using the method depicted in the standard of CLSI (2012). The minimum inhibitory concentration test was carried out in test tubes using the standard broth microdilution method, and the minimum bactericidal concentration test was performed on the nutrient agar plates. For the minimum inhibitory concentration test, 1 mL of the AgNPs stock solution (450 μg/mL) was serially diluted 3-fold with the nutrient broth media containing eight test tubes marked T1 to T8 with concentrations of 150 µg/mL (T1), 50 µg/mL (T2), 16.6 µg/mL (T3), 5.5 µg/mL (T4), 1.8 µg/mL (T5), 0.61 µg/mL (T6), 0.20 µg/mL (T7), and 0.06 µg/mL (T8); 30 µL of an overnight culture of *E. coli* (OD = 0.6) was added to all of these test tubes. The positive control was maintained without the addition of AgNPs. Test tube T1 contained the highest concentration of AgNPs, while test tube T8 showed the lowest concentration. The test tubes were incubated at 37 °C for 24 h. OD was measured at 600 nm to check the turbidity and the corresponding minimum inhibitory concentration. A minimum bactericidal concentration test was performed by streaking the suspension from each tube in a zigzag manner into a nutrient agar plate. The plates were incubated at 37 °C for 24 h. The lowest concentration with no visible growth on the nutrient agar plate was taken as a minimum bactericidal concentration value.

#### 2.6.2. Antifungal Assay by Disc Diffusion Method

The antifungal activity of AgNPs against blast pathogen of pearl millet (*Magnaporthe grisea*) was evaluated using Kirby Bauer antibiotic disc diffusion assay. *Magnaporthe grisea* was subcultured on oatmeal agar medium and incubated at 23 ± 2 °C for 10 days. Sterile discs impregnated with double distilled water and fluconazole were used as negative and positive controls, respectively. Different concentrations of AgNPs (5, 10, and 20 µg/µL) were added to the discs and incubated at room temperature for 10 days. Antifungal activity was measured based on the zone of inhibition around the disc impregnated with double distilled water, fluconazole, or AgNPs.

#### 2.6.3. Preparation of Platelets

Blood was collected from healthy human volunteers with informed consent according to the approved guidelines of the Institutional Human Ethical Committee (IHEC-UOM number 114 Ph.D/2015–16), University of Mysore, Mysore. Blood was drawn from an antecubital vein and was immediately mixed with acid citrate dextrose (ACD) anticoagulant, and platelets were isolated as described earlier [20]. Briefly, human platelet-rich plasma (PRP) was prepared by centrifuging anti-coagulated blood at 150× *g* for 15 min and the supernatant was collected and centrifuged at 700× *g* for 10 min at 37 °C. The platelet pellet was washed twice by suspending them in CGS (13 mM trisodium citrate, 33 mM D-glucose, 123 mM NaCl, pH 6.5) buffer and centrifuged at 700× *g* for 15 min at 37 °C. Finally, the washed platelets (WPs) were suspended in Tyrode’s buffer (2.5 mM HEPES, 150 mM NaCl, 2.5 mM KCl, 12 mM NaHCO_3_, 1 mM CaCl_2_, 1 mM MgCl_2_, 5.5 mM D-glucose, pH 7.4). The cell count was determined in WPs suspension using a Neubauer chamber and adjusted to 2 × 10^7^ platelets/mL in the final suspension using Tyrode’s buffer [21].

#### 2.6.4. Evaluation of Platelet Viability by MTT Assay

MTT colorimetric assay was performed to assess the platelet viability. Washed platelets (1 × 10^6^ cells/mL) were taken separately in 96-well microtiter plates. We treated the platelets with different concentrations of diluted AgNPs, specifically 0.5, 1, 1.5, 2, and 2.5 µg/µL, with 1× PBS followed by incubation for 2 h at 37 °C. Then after the incubation, 250 μM of MTT (3- (4,5-dimethylthiazol-2-yl)-2,5-diphenyltetrazolium bromide) was added and incubated for an additional 3 h. H_2_O_2_ was taken as the positive control. Thereafter, MTT was removed, and remaining formazan crystals were completely dissolved in DMSO and the absorbance was recorded at 570 nm using a Varioskan multimode plate reader (Thermofischer scientific, Waltham, MA, USA).

#### 2.6.5. Isolation of Human Erythrocytes/RBCs

Erythrocytes were isolated from the blood obtained from healthy human donors provided with informed consent, in accordance with the guidelines of institutional Human Ethical Committee (IHEC-UOM number 112/Ph.D/2015-16) guidelines, University of Mysore, Mysore. Freshly drawn blood was anti-coagulated with ACD and centrifuged to pellet the erythrocytes.

#### 2.6.6. Hemolysis Assay

Erythrocytes (5% hematocrit) in Ringer solution ((in mM) 125 NaCl, 5 KCl, 1 MgSO_4_, 32 N-2hydroxyethylpiperazine-N-2-ethane sulfonic acids (HEPES), 5 glucose, 1 CaCl_2,_ pH 7.4)) were incubated independently with indicated concentrations of diluted AgNPs (0.5, 1, 1.5, 2, and 2.5 µg/µL) with 1× PBS followed by incubation for 6 h at 37 °C. The absorbance of the supernatant was measured against blank at 415 nm for hemoglobin leakage (Beckman coulter DU-730, Brea, CA, USA). Untreated erythrocytes lysed in distilled water represent 100% hemolysis [22].

## 3. Results and Discussion

### 3.1. Isolation and Identification of Endophytic Bacteria from Terminalia arjuna

**Figure 1:**
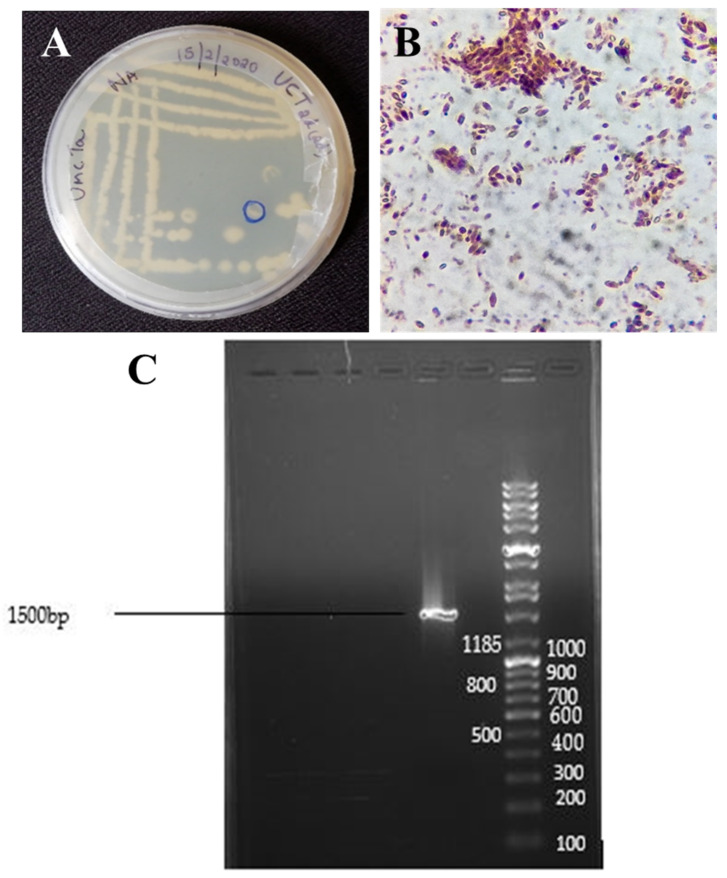
(**A**) Pure culture of isolated Endophytic bacteria from the bark samples. (**B**) Gram staining of isolated endophytic bacteria. (**C**) Agarose gel electrophoresis of the PCR product obtained from the 16SrRNA gene from endophytic bacteria.

The endophytic bacterial species from the bark of *Terminalia arjuna* was evaluated using LB medium. Surface sterilization protocol was followed and observed effectiveness. The sample was rinsed with water; it showed no microbial growth on LB medium after incubation at 37 °C for 10–15 days. Indeed, the most important key to being successful in isolating and studying endophytes is to ensure the appropriate sterility of the plant surface. After the surface sterilization of bark samples, individual colonies are separated using the spread plate method. The pure culture of the desired colony was selected (Figure 1A). Gram staining results showed that the endophytic bacteria are gram-positive with an oval shape (Figure 1B).

### 3.2. Molecular Identification of Endophytic Bacteria

The bacterial isolate was further characterized by 16S rRNA sequencing using PCR. The results of PCR amplification revealed that the size of the PCR product was 1500 bp (Figure 1C). Using 16S rRNA gene sequence data, we have interpreted that the nearly full length of the 16S rRNA gene from the endophytic bacteria possessed 96.71% similarity with *Cytobailus firmus* (Accession ID: NR_112635.1) that has been described in the GenBank database.

### 3.3. Screening of Cytobacillus firmus for the Synthesis of Silver Nanoparticles

**Figure 2:**
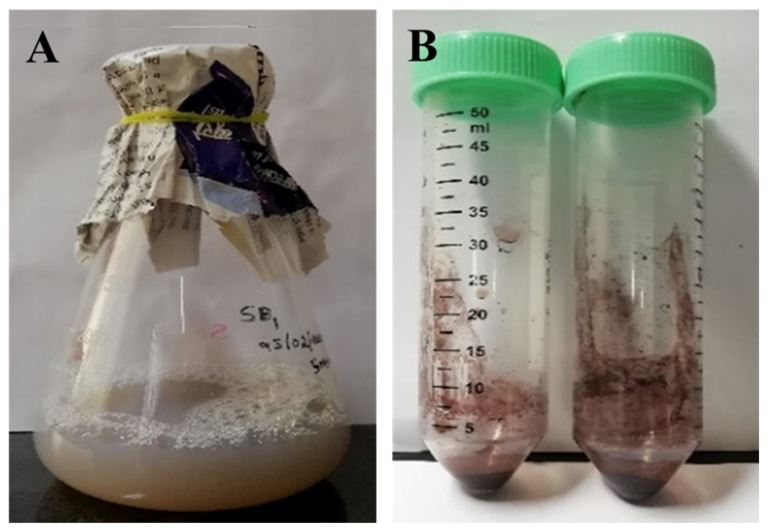
(**A**) Bacterial culture impregnated with metal Ag. (**B**) Green AgNPs (5 mM) are synthesized by endophytic bacteria from the bark of *Terminalia arjuna*.

The reduction of silver nitrate by the endophytic bacteria was seen by the color change observed from whitish to yellowish-brown, as shown in Figure 2A,B. Validation of the development of AgNPs was confirmed by the appearance of an SPR resonance peak between 300–600 nm.

### 3.4. Physical Characterization

#### 3.4.1. UV–Visible Spectroscopy

**Figure 3:**
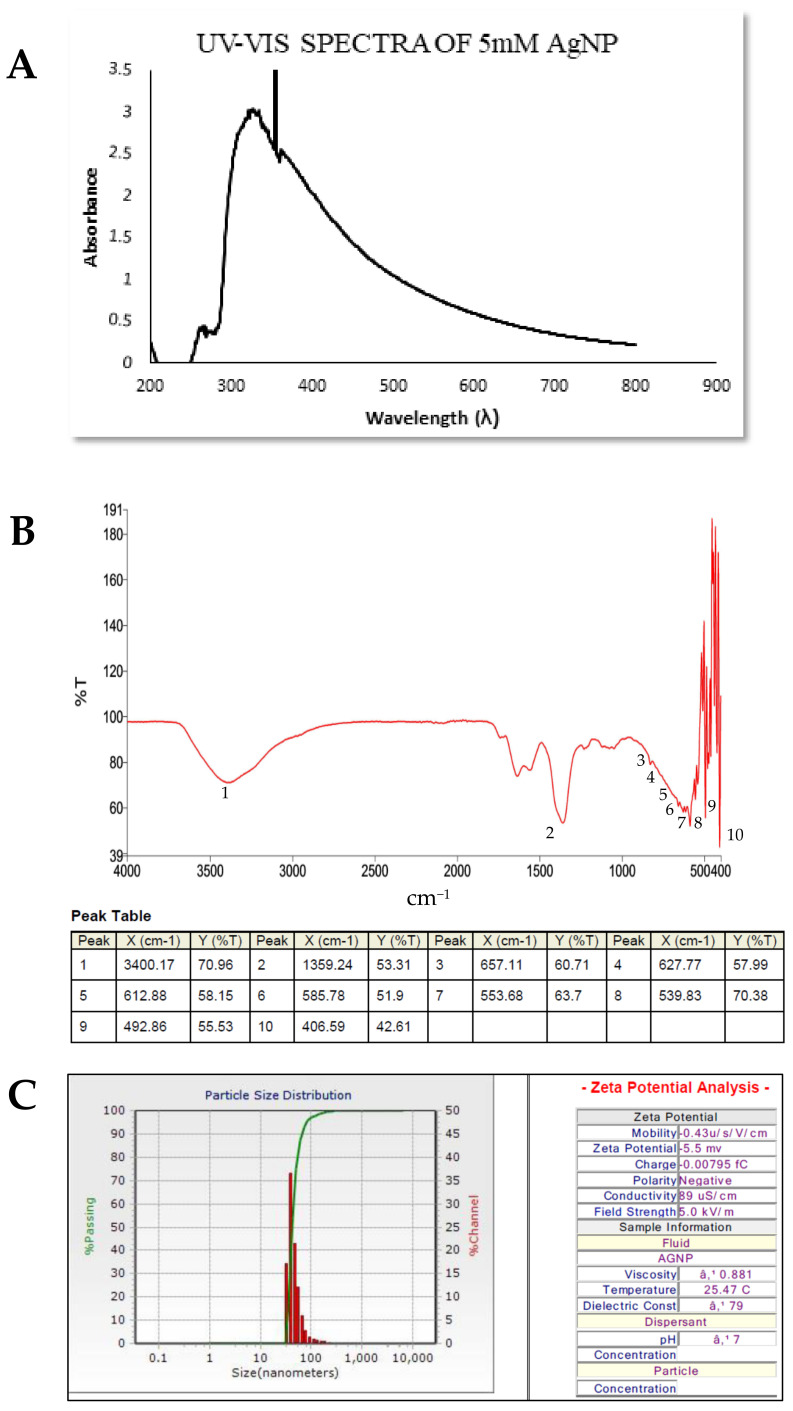

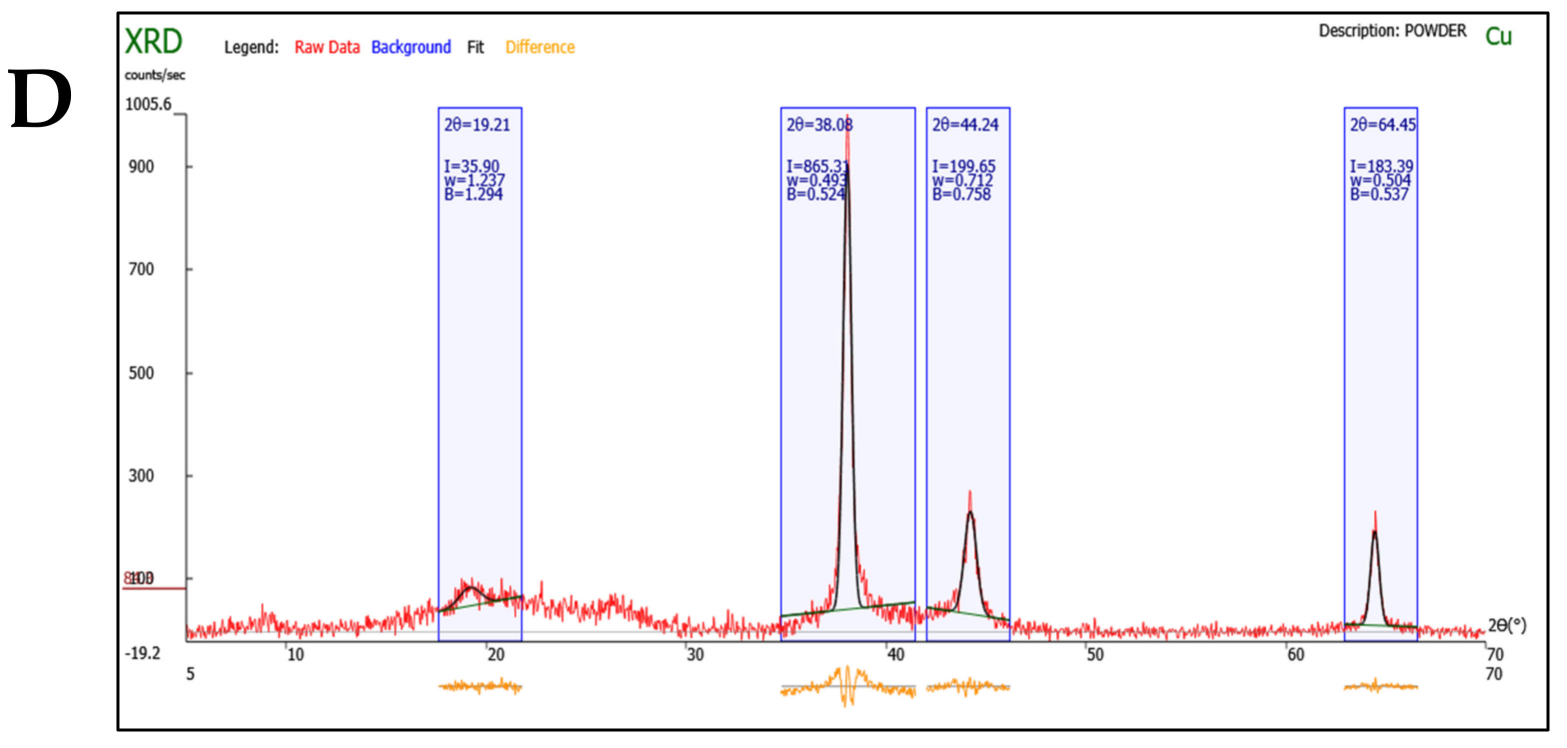
(**A**) UV–Visible spectroscopy of AgNPs showing peaks between 300 and 400 nm. (**B**) FTIR spectrum of synthesized AgNPs. (**C**) Dynamic light scattering analysis of AgNPs with varying sizes. (**D**) XRD diffraction pattern of silver nanoparticles synthesized from endophytic bacteria from *Terminalia arjuna.*

Characterization of biosynthesized AgNPs was accomplished using UV–Vis spectroscopy. In the UV–Visible spectrum, a strong broad peak was observed at 320 nm (Figure 3A), endorsing the presence of AgNPs, and widening of the peak indicated that the particles were polydispersed. AgNPs are proven to exhibit a UV–Visible absorption maximum in the range of 300–600 nm because of surface plasmon resonance (SPR). AgNPs have free electrons, which provide SPR absorption band by the mutual vibration of electrons of metal nanoparticles in resonance with the light wave.

#### 3.4.2. Fourier Transform Infrared Spectroscopy (FTIR)

FTIR is a key tool for the analysis of functional groups that are involved in the stabilization of synthesized AgNPs. The FTIR spectrum shown in Figure 3B of green AgNPs discloses clear peaks throughout the whole range of observation. FTIR analysis showed visible bands at 3400.17 cm^−^^1^, 1359.24 cm^−^^1^, 657.11 cm^−^^1^, 627.77 cm^−^^1^, 612.88 cm^−^^1^, 585.78 cm^−^^1^, 553.68 cm^−^^1^, 539.83 cm^−^^1^, 492.86 cm^−^^1^, and 406.59 cm^−1^ for synthesized AgNPs. The band found at 3400.17 cm^−^^1^can be assigned to O–H (alcohol) and/or N–H (amine) stretching. The band seen at 1359.24cm^−^^1^ is attributed to S=O (sulphonamide) stretching. The peaks observed at 657.11 cm^−^^1^, 627.77 cm^−^^1^, and 612.88 cm^−^^1^ correspond to C–Br (halo compound) stretching, whereas peaks at 585.78 cm^−^^1^, 553.68 cm^−^^1^, and 539.83 cm^−^^1^ represent both C–Br and C–I stretching (halogens). The FTIR data indicate that the biological molecules could be involved in both the synthesis and stabilization of AgNPs.

#### 3.4.3. Dynamic Light Scattering (DLS)

The DLS size distribution image of biosynthesized AgNPs is shown in Figure 3C. The size distribution of AgNPs ranges from 36 nm to 687 nm. The calculated average particle size distribution of AgNPs is 42.2 nm. The zeta potential of the biosynthesized AgNPs was found as a sharp peak at –5.5 mv, which implies that the surface of the nanoparticles was negatively charged and they were uniformly spread in the medium.

#### 3.4.4. XRD Analysis

Analysis of the structure and crystalline size of the synthesized silver nanoparticles was carried out by XRD. The XRD analysis of silver nanoparticles synthesized by *Cytobacillus firmus* showed diffraction peaks at 19.21°, 38.08°, 44.24°, and 64.45° (Figure 3D). When compared with the standard, the obtained XRD spectrum confirmed that the synthesized silver nanoparticles were in nanocrystal form and crystalline in nature. The peaks, 38.08°, 44.24°, and 64.45° can be assigned to the planes (111), (200), and (220) respectively—facets of the silver crystal. The same result was reported by Roy et al. and indicates that the silver nanoparticles are face-centered, cubic, and crystalline (correlated with JCPDS card: number 04-0783). The full width at half maximum (FWHM) values was used to calculate the sizes of the nanoparticles. The average size of silver nanoparticles synthesized from endophytic bacteria was calculated using Scherrer’s equation where Scherrer’s constantvalue of 0.94 was selected due to the cubic and crystalline nature of the nanoparticles. The average size of the synthesized nanoparticles from endophytic bacteria was found to be 14.23 nm.

#### 3.4.5. Scanning Electron Microscopy (SEM)

**Figure 4:**
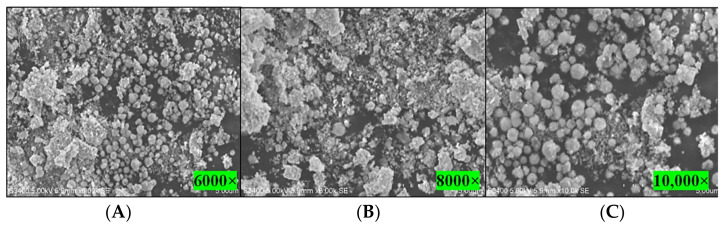
Scanning electron microscopy micrographs of synthesized silver nanoparticles at different magnifications: (**A**) 6000×, (**B**) 8000×, and (**C**) 10,000×.

SEM was employed to visualize the size and shape of silver nanoparticles. It is seen that AgNPs of spherical shape were obtained in bacterial extract being used as both a reducing and a capping agent (Figure 4A–C). This may have been due to the accessibility differences and the presence of various capping and reducing agents present in the bacterial extract. The development of AgNPs and their morphological characteristics in the SEM study at different magnifications, specifically at 6000×, 10,000×, and 8000×, verified that the average size was between 30 and 45 nm with inter-particle distance.

### 3.5. Biological Studies

#### 3.5.1. Fluorescent Microscopy

**Figure 5:**
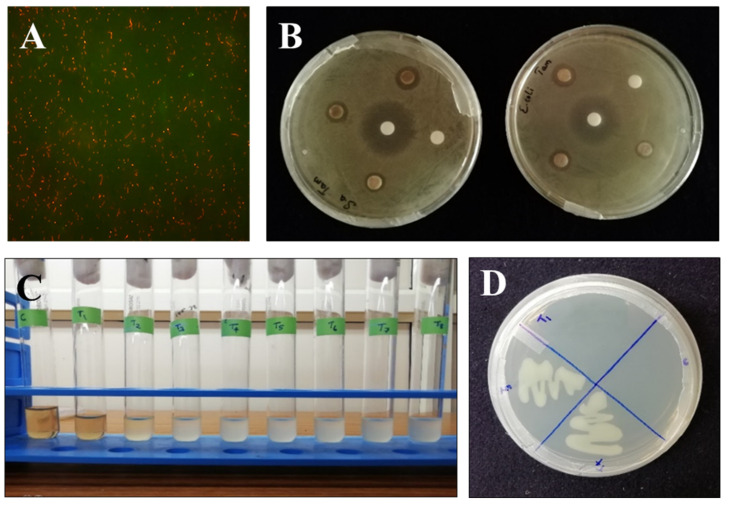
(**A**) Antimicrobial activity of AgNPs against *Escherichia coli* using fluorescent microscopy. (**B**) Antibacterial activity of different concentrations (5, 10, and 20 µg/µL) of AgNPs against *Staphylococcus aureus* and *Escherichia coli* using the disc diffusion method. (**C**) Determination of minimum inhibitory concentration (150 µg/mL) of AgNPs showing antibacterial activity. (**D**) The minimum bactericidal concentration of AgNPs.

To determine whether AgNPs induce strong toxicity to bacteria and verify the reliability of the bactericidal experiment, we conducted experiments using acridine orange (AO) and ethidium bromide (EtBr). AO and EtBr are effective imaging dyes for the elucidation of cell membrane damage. Live bacterial cells have intact membranes and are impermeable to AO, which can only pass into cells with disconcerted membranes. However, EtBr, a membrane-permeable dye, can easily cross the bacterial membrane and associate with DNA in the cell nucleus, emanating green fluorescence. Here we confirmed the antibacterial activity of synthesized AgNPs against *Escherichia coli*. A noteworthy uptake of green fluorescence by untreated *Escherichia coli* cells were observed using a fluorescence microscope (Carl Zeiss). However, after counterstaining with AO, both bacterial cells incubated with AgNPs exhibited red fluorescence (Figure 5A) as a result of the influx of membrane-impermeable fluorescent AO, indicating that the membrane integrity of the bacteria was disturbed, showing the effective antibacterial activity of AgNPs.

#### 3.5.2. Antibacterial Test Using Disc Diffusion Method

**Figure 6:**
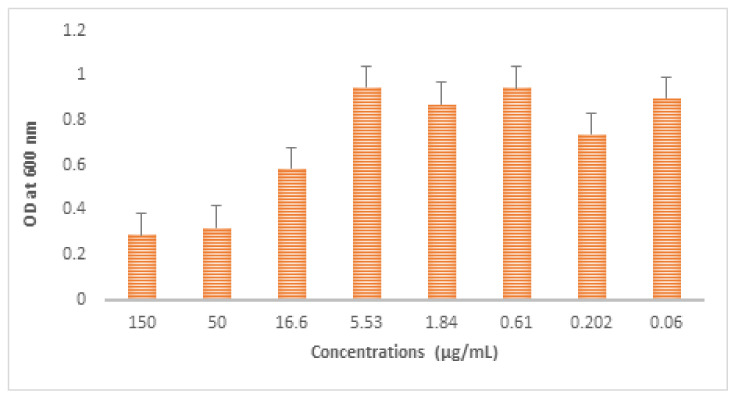
Graph showing different OD values obtained from different concentrations of AgNPs against *Escherichia coli.*

The antibacterial activities of the AgNPs were investigated against *Escherichia coli* and *Staphylococcus aureus* using the agar disc diffusion method as described earlier [23]. The diameters of the zones of inhibition for different concentrations of AgNPs were measured and expressed in millimeters (Table 1). In the present study, the diameters of the zones of inhibition were found to be 18 mm, 15 mm, and 12mm at 20 µg/µL, 10 µg/µL, and 5 µg/µL, respectively. The antibacterial activity of AgNPs was found to increase with the increase in the volume of AgNPs (Figure 5B). Similarly, the zone of inhibition was obtained against *Staphylococcus aureus* and the results are shown in Table 1. The minimum inhibitory concentration values of AgNPs were determined as described in the methods section and the results are presented in Table 2. Different concentrations of AgNPs were prepared (Figure 5C) and the corresponding absorbance was measured using UV–Visible spectroscopy at 600 nm. The concentration of AgNPs required to inhibit bacteria was 150 µg/mL. The minimum concentration of AgNPs as an antibacterial agent needed to execute bacteria was determined by minimum bactericidal concentration (MBC). Concentrations of AgNPs that are nearer to minimum inhibitory concentration (MIC) were tested for their minimum bactericidal concentration (MBC). The concentration of AgNPs which was identical to its minimum inhibitory concentration [(MIC 150 µg/mL)] was proven to provide effective bactericidal activity (Figure 5D), whereas the concentrations tested other than the minimum inhibitory concentration (MIC) (Figure 6) were not ideal for a bactericidal effect.

#### 3.5.3. Antifungal Activity of AgNPs against *Magnaporthe grisea*

**Figure 7:**
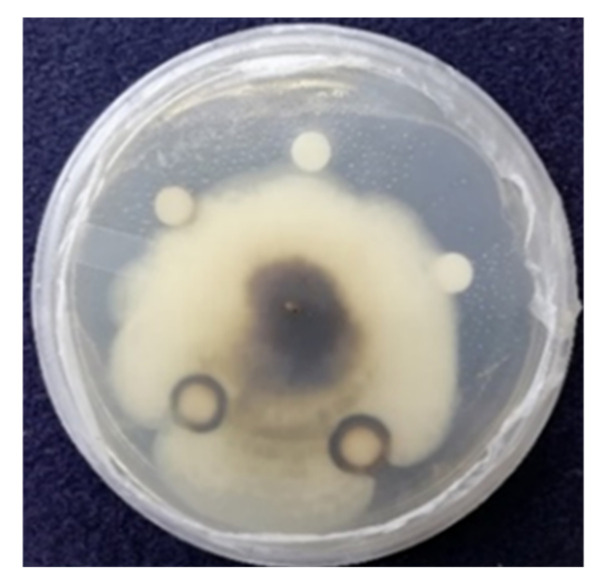
Antifungal activity of AgNPs against *Magnaporthe grisea* by disc diffusion method.

The antifungal activity of the synthesized AgNPs was investigated against blast pathogen *Magnaporthe grisea* using the agar disc diffusion method. The diameters of the zones of inhibition (in mm) for different volumes of AgNPs were measured. The volumes of AgNPs added to the discs were 5 µg/µL, 10 µg/µL, and 20 µg/µL. The highest zone of inhibition was found for 20 µg/µL (16 mm), whereas the disc loaded with 10 µg/µL of AgNPs showed a diameter of 14 mm. No inhibition zones were visible for 5 µg/µL and negative control (water) (Figure 7). These results suggested that the synthesized AgNPs are a potent antifungal agent.

#### 3.5.4. Evaluation of Platelet Viability by MTT Assay

**Figure 8:**
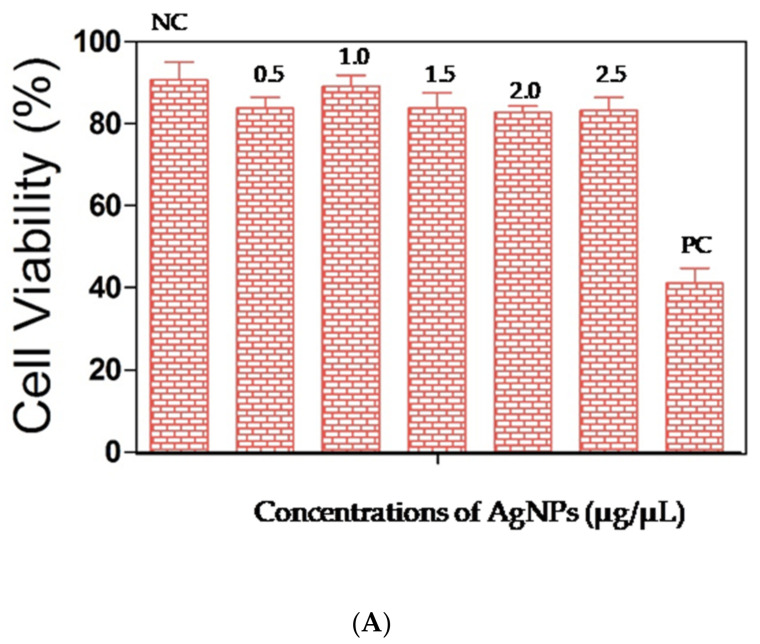

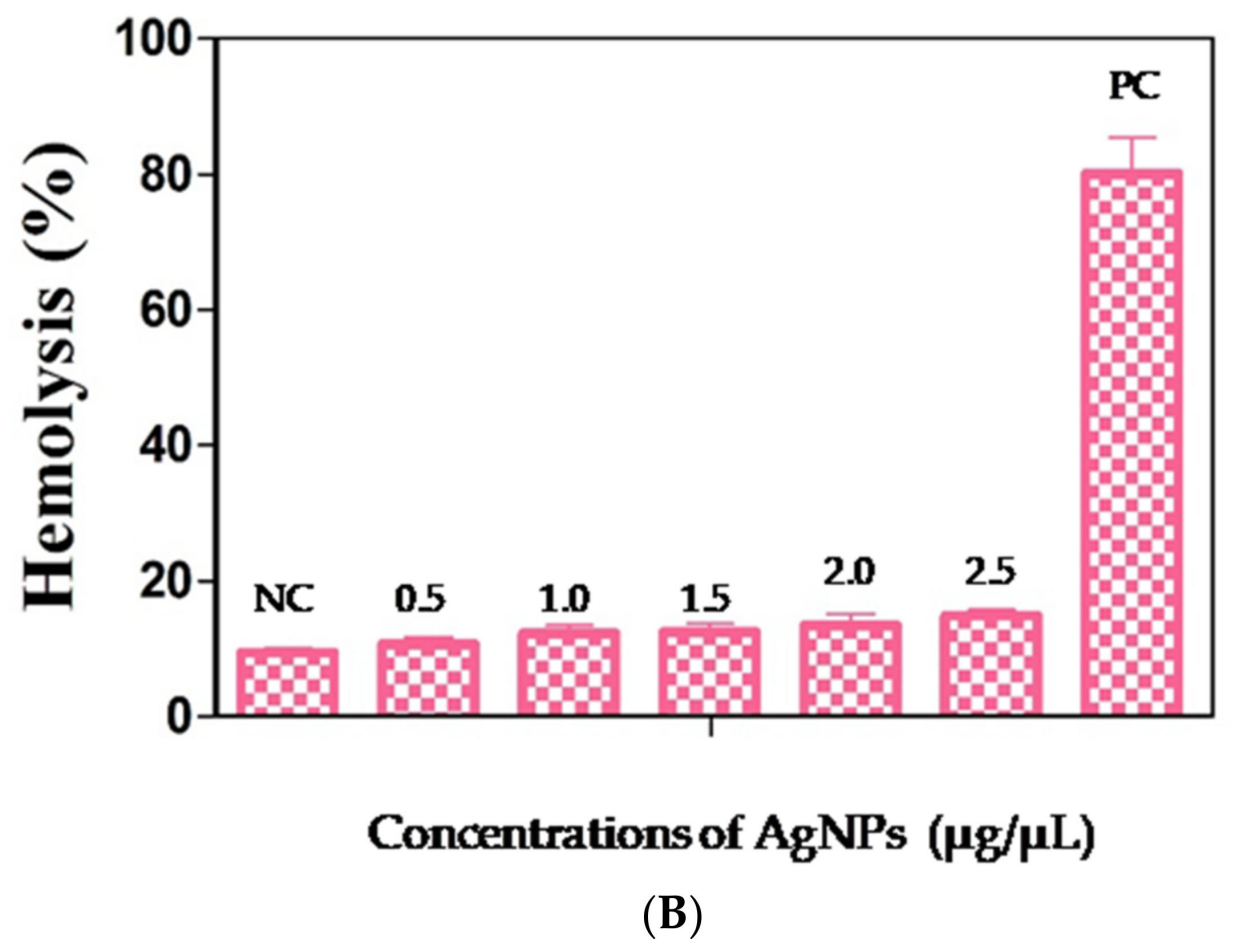
(**A**) Cell viability assay of AgNPs on human platelets. (**B**) Hemolysis assay of AgNPs on human erythrocytes.

The cytotoxic potential of AgNPs was evaluated on platelets using formazan formation assay (MTT assay). The percentage of viable cells was significantly higher in the AgNPs treated cells (≈80–90%) compared with the cells that were treated with hydrogen peroxide (40%)(positive control (PC)) (Figure 8A): 1 µg/µL of AgNPs exactly had shown the same percentage of cell viability as a negative control (≈90%), whereas the degrees of cell viability exhibited at concentrations, 0.5 µg/µL, 1 µg/µL, 1.5 µg/µL, 2 µg/µL, and 2.5 µg/µL of AgNPs were consistent at 80%, indicating that these AgNPs are not involved in platelet death.

#### 3.5.5. Hemolysis Assay on Human Erythrocytes

We performed a hemolysis assay to investigate the effect of AgNPs on erythrocytes. For this, we measured the hemoglobin release upon AgNPs treatment using a spectrophotometer at 415 nm. The extent of hemolysis with AgNPs was compared with the erythrocytes lysed in distilled water (positive control, PC) and human erythrocyte cells alone (negative control, NC). AgNPs did not induce hemolysis at different concentrations and the extent of hemolysis was similar to the negative control (Figure 8B). The percentages of hemolysis caused by AgNPs at 0.5 µg/µL, 1 µg/µL, 1.5 µg/µL, 2 µg/µL, and 2.5 µg/µL were found to be nearly 10%, which is the same as the negative control. The erythrocytes lysed in distilled water (PC) showed a maximum degree of hemolysis of ≈80%.

## 4. Conclusions

The present study indicated the green synthesis of silver nanoparticles by a biological method using *Cytobacillus firmus* that supply both reducing and stabilizing agents for the biosynthesis of nanoparticles. The silver nanoparticles were characterized further by DLS, SEM, and FTIR spectroscopy. The roles of endophytic bacterial secondary metabolites for the metal reduction, stabilization, and capping of silver nanoparticles were qualitatively analyzed. The biosynthesized AgNPs have potent antimicrobial activity at lower concentrations for both human and plant pathogens. Moreover, cytotoxicity assays revealed that the new AgNPs did not induce toxicity towards either platelets or erythrocytes, indicating that these biogenic nanoparticles are non-toxic. Besides, they may be used as a promising anti-bactericidal and fungicidal compound in the food, medicine, and agriculture sector.

## Figures and Tables

**Table 1:** Table showing different volumes of AgNPs used against *Escherichia coli* and *Staphylococcus aureus* in the disc diffusion method.

Concentrations of AgNPs (µg/µL)	Zone of Inhibition (mm)	
	*Escherichia coli*	*Staphylococcus aureus*
5	12	11
10	15	15
20	18	20
Streptomycin	24	23

**Table 2:** Different concentrations of AgNPs were used and their corresponding OD values.

Test Samples	Concentration of AgNPs (µg/mL)	OD Values (600 nm)			Mean	Standard Deviation	Mean + SD
T1	150	0.099	0.097	0.099	0.098	0.1918	0.289
T2	50	0.150	0.153	0.152	0.151	0.1717	0.322
T3	16.6	0.573	0.575	0.575	0.574	0.0118	0.585
T4	5.53	0.855	0.855	0.857	0.855	0.0938	0.948
T5	1.84	0.810	0.790	0.800	0.8	0.0728	0.872
T6	0.61	0.851	0.853	0.852	0.852	0.0927	0.944
T7	0.202	0.690	0.720	0.700	0.703	0.0360	0.739
T8	0.06	0.818	0.820	0.818	0.818	0.08	0.898

## Data Availability

The data presented in this study are openly available.
